# Cultivated meat could aid in reducing global antimicrobial resistance burden – producing meat without antibiotics as a safer food system for the future

**DOI:** 10.1097/JS9.0000000000000199

**Published:** 2023-02-16

**Authors:** AbdulRahman A. Saied, Deepak Chandran, Hitesh Chopra, Abhijit Dey, Talha B. Emran, Kuldeep Dhama

**Affiliations:** a National Food Safety Authority (NFSA), Aswan Branch, Aswan, Egypt; b Ministry of Tourism and Antiquities, Aswan Office, Aswan, Egypt; c Department of Veterinary Sciences and Animal Husbandry, Amrita School of Agricultural Sciences, Amrita Vishwa Vidyapeetham University, Coimbatore, Tamil Nadu, India; d Chitkara College of Pharmacy, Chitkara University, Rajpura, Punjab, India; e Department of Life Sciences, Presidency University, Kolkata, West Bengal, India; f Division of Pathology, ICAR-Indian Veterinary Research Institute, Bareilly, Uttar Pradesh, India; g Department of Pharmacy, Faculty of Allied Health Sciences, Daffodil International University, Dhaka, Bangladesh; h Department of Pharmacy, BGC Trust University Bangladesh, Chittagong, Bangladesh

Highlights

- Widespread use of antibiotics in livestock production contributes to public health concerns.
- Animal-origin foods could be contaminated with antimicrobial resistance (AMR) bacteria.
- AMR bacteria and genes have been found in various products of animal origin.
- Therapeutic expenses have increased in rich countries due to AMR bacteria-caused infections.

*Dear Editor*,

It is well accepted that the widespread use of antibiotics in livestock production contributes to the worldwide public health concern known as antimicrobial resistance (AMR). Antibiotics are widely utilized in the cattle business as growth enhancers and as a cheap alternative to good sanitary practices. The intensive use of antibiotics in animal husbandry has been identified as a major contributor to AMR by the United Nations General Assembly 1. Animals in crowded settings are more likely to be exposed to disease-causing pathogens and experience stress, leading farmers to overuse antibiotics to keep their livestock alive and continue producing meat. Meat from wild and domesticated animal and fish sources has been found to have antibiotic residues.

The use of antibiotics in intensive animal husbandry and aquaculture practices has a secondary impact on the local ecosystem. AMR genes can proliferate in aquaculture systems that could be exposed to manure runoff from animal agriculture. Eliminating needless antibiotic use in food production could lessen the threat of rising AMR. Countries that export items that do not require using antibiotics could benefit economically and public health-wise if they were given incentives. Examples of such efforts include European regulatory measures aimed at reducing the overuse of antibiotics in animal husbandry, but due to a lack of worldwide surveillance, enforcing such restrictions consistently has proven difficult.

Animal-origin foods could be contaminated with AMR bacteria (AMRB). *Salmonella*, often found in contaminated poultry, pork, and beef, has developed into a drug-resistant strain. Poultry act as a major vector for the spread of AMR *Campylobacter* 2 and turkey has been associated with AMR *Salmonella* 3,4. AMRB and genes have been found in various products of animal origin, such as plasmid-mediated tigecycline-resistance genes, tet(X3) and tet(X4), in various bacterial species isolated from animals, meat for consumption, and humans 5 and plasmid-mediated colistin-resistance genes (mcr) 6.

Resistance to antibiotics with essential medicinal uses is being fueled by their unjudged usage in livestock and aquaculture production. In order to fulfill the rising demand for protein, methods for producing meat without antibiotics will be necessary, as global regulation alone is unlikely to be sufficient to control antimicrobial use. One way to separate antibiotics from meat is to produce it in different ways, such as cultured meat (CM) 7,8. In November 2022, Upside Foods Company, a San Francisco-based start-up, got approval from the Food and Drug Administration for producing CM. The company will be able to sell chicken made from real animal cells grown in bioreactors instead of slaughtering live animals. The next steps and procedures are expected to be swift, and these meats produced by Upside Foods Company will be available to the public in the US market.

There are three pivotal questions: Could the CM solve part of AMR? Theoretically, cultivated meat could be free from drug residues, resistant bacteria, and food-borne illness pathogens. Compared to the current meat business, the use of antibiotics in the preproduction stage of CM will be extremely low, in addition to the tendency of CM producers to eliminate antibiotic usage in their processes 9,10. Singapore is now the only country where these meats are lawfully sold to consumers. In the coming months, the Food and Drug Administration’s approval of Upside Foods’ cultivated meat as safe to consume is likely to unleash a wave of different kinds of cultivated meat into the United States.

Therapeutic expenses have increased in rich countries due to AMRB-caused infections, but the increased rates of related morbidity and death are shown mainly in developing countries. Given that CM is made in a laboratory, it is protected from any potential contamination that may occur during the slaughter of animals. Traditional animal products are a major contributor to the epidemic, food-borne disease, and food recalls; hence, CM is promoted as safer. *Salmonella* and *Escherichia coli* are found in the gut and can contaminate the food supply and cause food poisoning if they are excreted. These will not be present in a cultivated meat production facility.

Antimicrobial resistance is currently the largest cause of death worldwide, contributing to an estimated 4.95 million deaths annually. It is predicted that there will be 10 million AMR-related deaths annually by 2050. By 2050, the global population is expected to surpass 9.5 billion, with the expected increased demand for meat to be 73%. So, we must design a food system for the future that will simultaneously protect the planet’s natural resources and biodiversity while also providing people with access to cheap, nutritious food. This situation calls for a long-term solution, and CM could provide that, by guaranteeing contaminants-free procedures during the stages of manufacturing of CM and animal-component-free commercial production.

## Ethical approval

Not applicable.

## Sources of funding

None.

## Author contributions

A.A.S.: conceptualization, data curation, writing – original draft preparation, writing – reviewing and editing. D.C., H.C., A.D., and K.D.: data curation, writing – original draft preparation, writing – reviewing and editing. T.B.E.: writing – reviewing and editing, visualization, supervision.

## Conflicts of interest disclosure

Authors declare that they have no conflicts of interest.

## Research registration unique identifying number (UIN)

None

## Guarantor

Talha Bin Emran, PhD, Associate Professor, Department of Pharmacy, BGC Trust University Bangladesh, Chittagong 4381, Bangladesh. Tel: +880 303 356 193, Fax: +880 312 550 224. https://orcid.org/0000-0003-3188-2272.

## Data statement

The data in this correspondence article is not sensitive in nature and is accessible in the public domain. The data is therefore available and not of a confidential nature.

## Provenance and peer review

Not commissioned, internally peer-reviewed.
